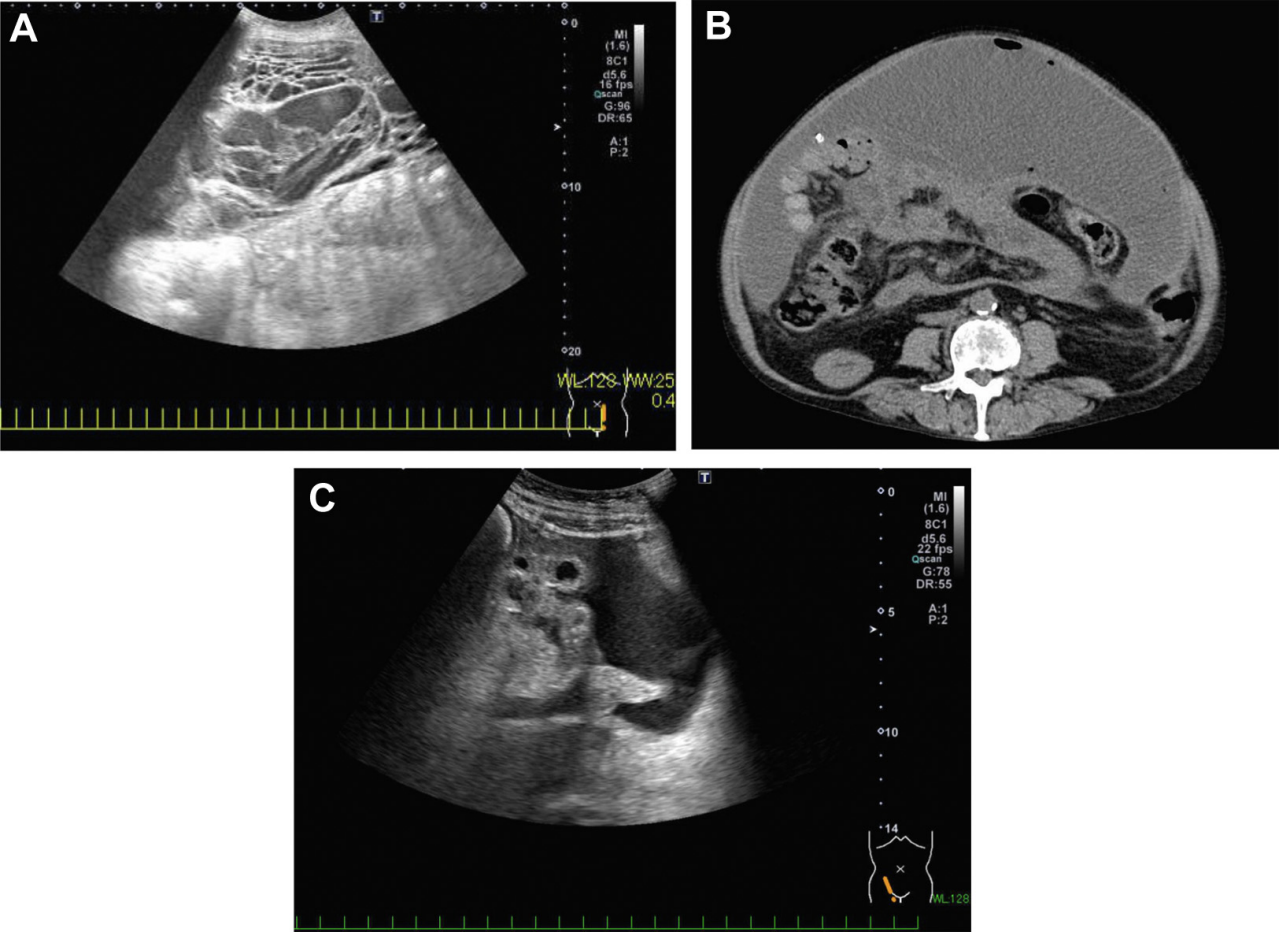# Ascites With Honeycombed Appearance

**DOI:** 10.1016/j.gastha.2022.09.010

**Published:** 2022-09-30

**Authors:** Kento Shionoya, Haruki Uojima

**Affiliations:** 1 Shonan Gastroenterology Medicine Center, Shonan Kamakura General Hospital, Kamakura, Kanagawa, Japan; 2 Department of Gastroenterology, Kitasato University School of Medicine, Sagamihara, Kanagawa, Japan

A 56-year-old woman presented with alcoholic cirrhosis with complications of ascites unless treated otherwise using diuretics (on furosemide, spironolactone, and tolvaptan) and ascites puncture. She had a history of alcoholic cirrhosis, obesity, and depression. A peritoneovenous shunt temporarily improved the ascites. Shunt obstruction worsened her abdominal distention a few weeks later. During the follow-up period, she had abdominal pain with a low-grade fever. Abdominal ultrasonography revealed significant ascites with a honeycombed appearance (Figure A); however, abdominal computed tomography did not indicate the same result (Figure B). The cell count analysis of the ascitic fluid revealed bacterial peritonitis. The infected shunt was removed, assuming that it caused the honeycombed appearance. Subsequently, ascites and abdominal distension improved. Four months after the shunt removal and continuous administration of antibiotics, abdominal ultrasonography results revealed decreased ascites without a honeycombed appearance (Figure C). Peritoneovenous shunt automatically injects ascites into the jugular vein. It can help effectively reduce the number of ascites. Although it is often effective in cases where liver function is relatively well maintained, and there is no hepatic encephalopathy, it is prone to complications such as peritonitis and heart failure, and shunt obstruction can easily occur.

**Figure:**